# Two decades of medication administration errors related to pain management: Hospital admissions for nonopioid analgesics, antipyretics, and antirheumatics in Australia

**DOI:** 10.1097/MD.0000000000042893

**Published:** 2025-06-13

**Authors:** Asaleh El-Qasem, Abdallah Y. Naser, Alaa A. Alsharif

**Affiliations:** a Faculty of Pharmacy, University of Jordan, Amman, Jordan; b Department of Applied Pharmaceutical Sciences and Clinical Pharmacy, Faculty of Pharmacy, Isra University, Amman, Jordan; c Department of Pharmacy Practice, College of Pharmacy, Princess Nourah bint Abdulrahman University, Riyadh, Saudi Arabia.

**Keywords:** analgesics, antipyretics, Australia, medication administration error, nonopioids

## Abstract

The rate of medication poisoning is constantly increasing globally, increasing the number of hospital admissions and the burden on the health system. In Australia, nonopioid analgesics, antipyretics, and antirheumatics medications cause a high rate of accidental poisonings. This study aims to study hospital admissions trends related to medication administration errors (MAEs; poisoning by, adverse effect of and underdosing of) of nonopioid analgesics, antipyretics, and antirheumatics-related in Australia from 1998 to 2022. This ecological study used hospital admissions data from the National Hospital Morbidity Database. Population data were extracted from the Australian Bureau of Statistics dataset. We analyzed data based on overall trends, type of admissions, and demographics. A total of 1,61,597 hospital admissions MAEs for nonopioid analgesics, antipyretics, and antirheumatics were recorded during study time. The annual number of admissions increased by 43.6%, reflecting a 3.8% increase in the hospital admission rate. The main reason for admissions was MAEs of 4-aminophenol derivatives. Most episodes were overnight admissions. The incidences of admissions were higher among younger age groups and females. Medication administration errors of nonopioid analgesics, antipyretics, and antirheumatics-related hospital admissions rose significantly in Australia. The most common causes of admission were poisonings by 4-aminophenol and other nonsteroidal anti-inflammatory drugs. Admissions are higher in the age group below 20 years and in females. Raising public awareness about medication risks and implementing targeted interventions are required.

## 1. Introduction

Acute poisoning is a principal cause of mortality, morbidity, and emergency department admissions all over the world.^[1,2]^ Pharmaceuticals are frequently implicated in diverse forms of poisoning, whether associated with acts of deliberate self-poisoning, therapeutic errors, or accidental exposures.^[3]^ In high-income countries, pharmaceuticals are the most important cause of poisoning.^[4]^ This encompasses over-the-counter (OTC) medications and medications prescribed by healthcare professionals.^[3]^ Globally, the poisoning rate from OTC and prescription medications is continually increasing.^[5]^

Usually, enhancing patients’ independence in healthcare involves growing the use of OTC drugs, involving self-care, self-medication, and self-treatment. Nonetheless, this practice also carries some difficulties, such as occasional incidents of OTC drug poisoning, the potential for side effects due to the consumption of numerous medications (polypharmacy), and the possibility of masking health issues through temporary alleviation of symptoms.^[6]^

Across all human exposures, analgesics were identified as the primary reason for drug poisoning, according to the 2022 Annual Report from the American Association of Poison Control Centres.^[7]^ A prior study found that nonopioid analgesics, antipyretics, and antirheumatics were the predominant groups of medication responsible for hospital admissions associated with medication errors in the United Kingdom.^[8]^ In Malaysia, nonopioid analgesics, antipyretics, and antirheumatics accounted for the bulk (17.2%) of poisoning admissions.^[9]^

In the past decade, poisoning fatalities have doubled in Australia, becoming the fifth leading cause of years of potential life loss.^[10]^ In Australia, analgesics are the most common category of pharmaceutical substances that result in fatalities.^[11]^ Furthermore, according to estimations in Australia, hospitalizations related to medications cost the healthcare system Australian dollar (AUD)$1.4 billion yearly, resulting from about 2,50,000 admissions episodes. In 2010/2011, nonopioid analgesics, antipyretics, and antirheumatics medications were responsible for 14% of accidental pharmaceutical poisonings in Australia.^[12]^ Numerous factors may impact these admissions trends, such as changes in prescribing practices,^[13]^ socioeconomic status,^[14]^ and demographics.^[15]^ Apprehending these trends is imperative to implementing efficacious interventions to reduce poisonings related to these drugs, reduce hospital admissions due to them, and thus lessen the burden on healthcare resources. Hence, this research aims to study hospital admissions trends related to medication administration errors (MAEs; poisoning by, adverse effect of and underdosing of) of nonopioid analgesics, antipyretics, and antirheumatics-related in Australia from 1998 to 2022.

## 2. Materials and methods

### 2.1. Data sources

#### 2.1.1. National Hospital Morbidity Database

The National Hospital Disease Database (NHMD) is a National Hospital Data Collection component. This collection is managed by the Australian Institute of Health and Welfare and includes the core national hospital datasets.^[16]^ Data provided by state and territory health authorities in Australia are collected by the NHMD, an online database.^[17]^ NHMD-collected data comprises episode-level record sets from Australia’s public and private hospitals’ morbidity data collection systems. The data are based on the National Minimum Data Set (NMDS) for Admitted Patient Care, which comprises information on hospital treatments, length of stay, external causes for poisoning and injury, patient diagnoses, demographics, and administrative information. NMDS for admitted patient care refers to data collected regarding the medical services involved in hospital-admitted patients’ care within Australian hospitals. Hospital admission episodes about patient care across all public and private psychiatric and acute hospitals, drug and alcohol treatment centers, and standalone day hospital facilities are encompassed by the NMDS. However, NMDS does not cover all hospitals operated by the Australian Defence Force, correctional authorities, or Australia’s offshore territories.

#### 2.1.2. Australian Bureau of Statistics

We utilized the Australian Bureau of Statistics (ABS) to collect mid-year population data between 1998 and 2022, as follows: Historical population data^[18]^ to gather data between 1998 and 2016, while national, state, and territory population data^[19]^ to gather data between 2017 and 2022. As the nation’s official statistical agency, the ABS produces independent and dependable data.^[11]^

### 2.2. Study population

This study included all admission data related to MAEs (poisoning by, adverse effect of and underdosing of) of nonopioid analgesics, antipyretics, and antirheumatics-related in Australian private and public hospitals from 1998 to 2022. This dataset was extracted from principal diagnosis data cubes at NHMD.^[20]^ Medication administration errors of nonopioid analgesics, antipyretics, and antirheumatics hospital admission episodes were identified using the Tenth Revision of the International Statistical Classification of Diseases and Related Health Problems (ICD-10) code T39.

### 2.3. Data analysis

Study data were analyzed using the Statistical Package for Social Science Software, version 29. Categorical variables were presented as frequencies and percentages. Admission rates were presented with 95% confidence interval. The significance level was assigned as *P* value <.05. Pearson’s *χ*^2^ test of independence was used to examine the different in admission rates.

## 3. Results

### 3.1. Trends in admission related to medication administration errors of nonopioid analgesics, antipyretics and antirheumatics

Between 1998 and 2022, Australia recorded a total of 1,61,597 hospital admissions due to MAEs of nonopioid analgesics, antipyretics, and antirheumatics. The annual number of admissions increased by 43.6%, from 5026 in 1998 to 7215 in 2022. This surge reflects a 3.8% increase in the hospital admission rate, from 26.72 (95% confidence interval [CI] 25.98–27.46) per 1,00,000 persons in 1998 to 27.73 (95% CI 27.09–28.37) in 2022 (*P* > .05).

**Figure 1.**
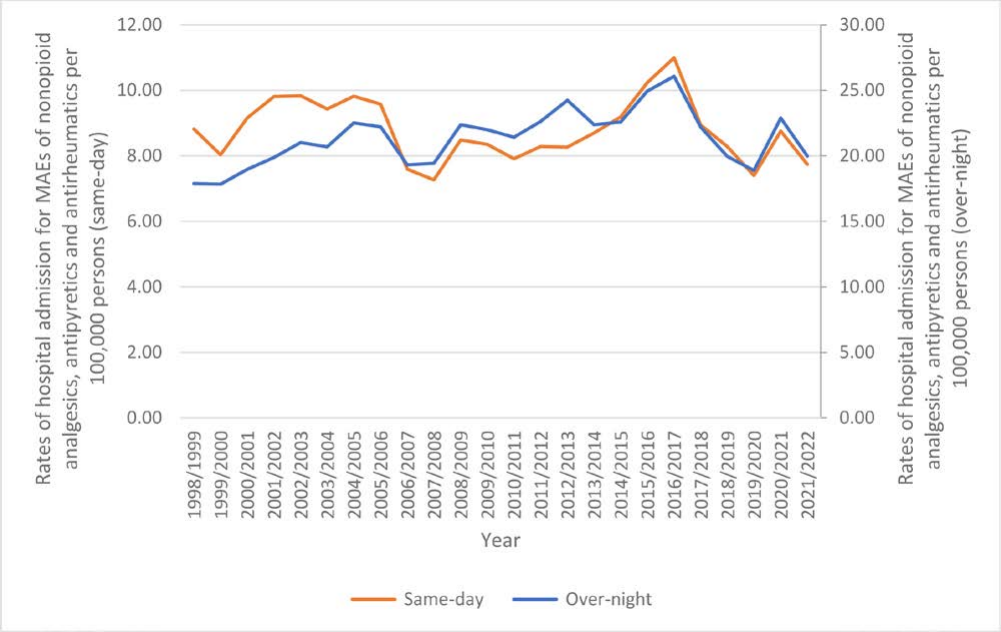
Rates of same-day and overnight-stay patients admission.

Overnight admissions comprised 71.0% of the total admissions, while 29.0% were same-day admissions. The rate of same-day admissions decreased by 12.3% from 8.82 (95% CI 8.39–9.24) per 1,00,000 persons in 1998 to 7.74 (95% CI 7.40–8.08) in 2022. Conversely, the rate of overnight-stay admissions increased by 11.7%, rising from 17.90 (95% CI 17.29–18.50) per 1,00,000 persons in 1998 to 19.99 (95% CI 19.44–20.53) in 2022 (Fig. 1).

The leading cause of MAEs admissions was MAEs of 4-aminophenol derivatives, which accounted for 83.5%, followed by other nonsteroidal anti-inflammatory drugs (NSAIDs), comprising 12.4% (Table 1).

**Table 1:** Percentage of MAEs stratified by therapeutic class.

ICD code	Therapeutic class	Percentage
T39.0	Salicylates	3.0%
T39.1	4-Aminophenol derivatives	83.5%
T39.2	Pyrazolone derivatives	˂0.1%
T39.3	Other nonsteroidal anti-inflammatory drugs [NSAID]	12.4%
T39.4	Antirheumatics, not elsewhere classified	0.3%
T39.8	Other nonopioid analgesics and antipyretics, not elsewhere classified	0.5%
T39.9	Nonopioid analgesic, antipyretic and antirheumatic, unspecified	0.3%

ICD = International Statistical Classification of Diseases System.

### 3.2. Change in admission rate stratified by therapeutic class

**Figure 2.**
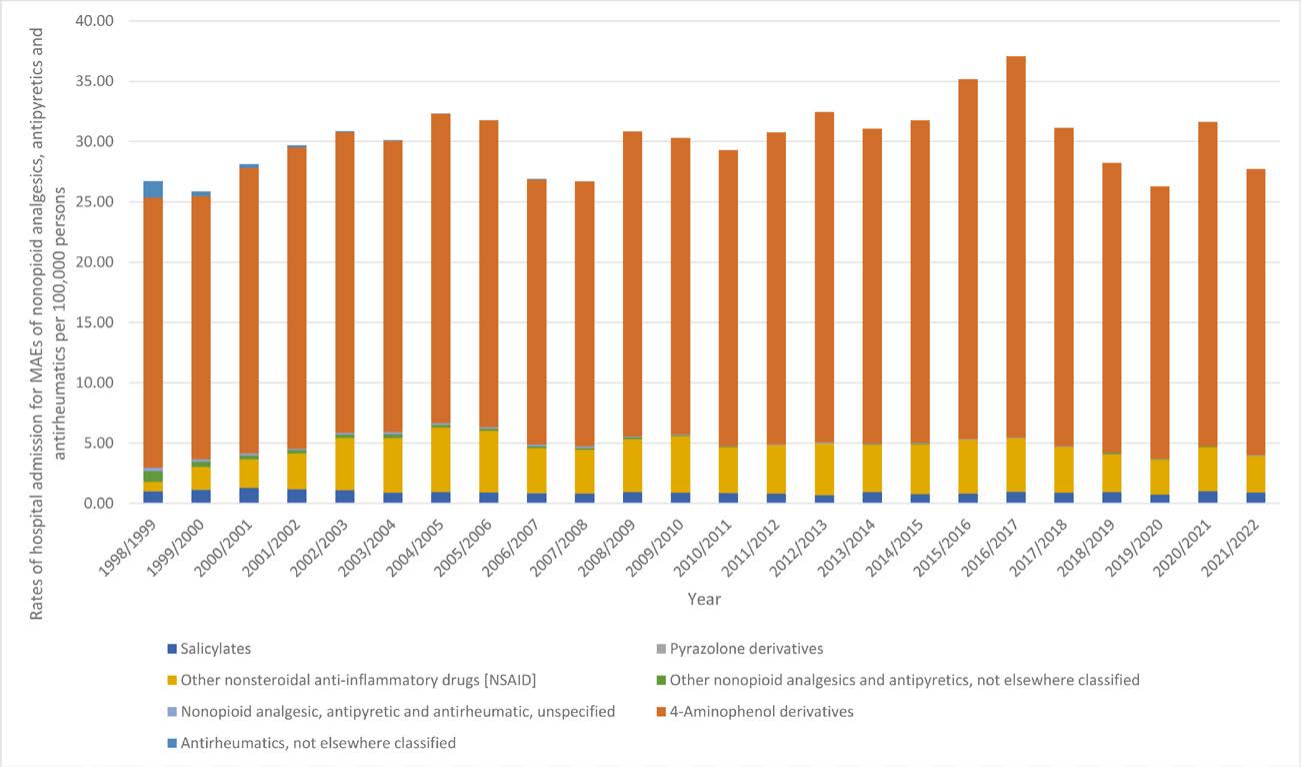
Admission rates stratified by type between 1998 and 2022.

During the study period, there was a significant increase in admission rates for MAEs of other NSAIDs, which rose by 2.91-fold. Additionally, admission rates for MAEs of 4-aminophenol derivatives increased by 6.0%. However, admission rates for MAEs of “antirheumatics, not elsewhere classified,” MAEs of pyrazolone derivatives, MAEs of other “nonopioid analgesics and antipyretics, not elsewhere classified,” MAEs of “nonopioid analgesic, antipyretic and antirheumatic, unspecified,” and MAEs of salicylates decreased by 100.0%, 100.0%, 97.4%, 88.7%, and 10.0%, respectively (Table 2, Fig. 2).

**Table 2:** Percentage change in admission rates for MAEs stratified by therapeutic class.

Poisonings	Rate of poisonings in 1998 per 1,00,000 persons (95% CI)	Rate of poisonings in 2022 per 1,00,000 persons (95% CI)	Percentage change from 1998 to 2022
Salicylates	1.00 (0.86–1.14)	0.90 (0.78–1.01)	−10.0%
4-Aminophenol derivatives	22.39 (21.72–23.07)	23.73 (23.14–24.32)	6.0%
Pyrazolone derivatives	0.01 (−0.01–0.02)	0.00 (0.00–0.00)	−100.0%
Other nonsteroidal anti-inflammatory drugs [NSAID]	0.78 (0.65–0.90)	3.04 (2.83–3.25)	291.3%
Antirheumatics, not elsewhere classified	1.37 (1.20–1.54)	0.00 (0.00–0.00)	−100.0%
Other nonopioid analgesics and antipyretics, not elsewhere classified	0.90 (0.76–1.03)	0.02 (0.00–0.04)	−97.4%
Nonopioid analgesic, antipyretic and antirheumatic, unspecified	0.27 (0.20–0.35)	0.03 (0.01–0.05)	−88.7%

NSAID = nonsteroidal anti-inflammatory drugs.

### 3.3. Trends in admissions stratified by gender

**Figure 3.**
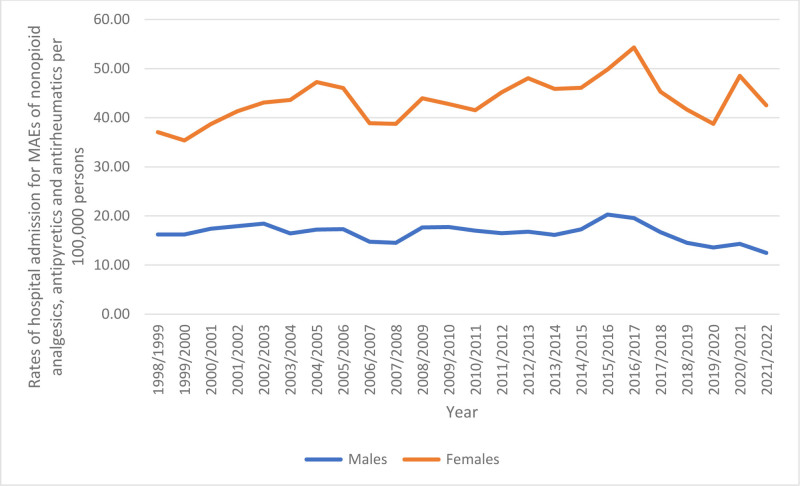
Admission rate stratified by gender.

Females accounted for 1,17,669 admission episodes due to MAEs of nonopioid analgesics, antipyretics, and antirheumatics, representing 72.9% of the total admissions, with an average of 4902.88 per year. The admission rate for MAEs decreased among males by 23.1%, from 16.23 (95% CI 15.41–17.05) per 1,00,000 persons in 1998 to 12.48 (95% CI 11.87–13.09) in 2022 (*P* < .001). Conversely, the admission rate for MAEs among females increased by 14.7%, rising from 37.06 (95% CI 35.83–38.28) per 1,00,000 persons in 1998 to 42.52 (95% CI 41.40–43.64) in 2022 (*P* < .001; (Fig. 3).

### 3.4. Trends in admissions stratified by age

In terms of age group distribution for rate of hospital admissions due to MAEs of nonopioid analgesics, antipyretics, and antirheumatics, individuals aged below 20 years accounted for 36.2% of the total admissions, followed by those aged 20 to 39 years with 33.8%, individuals aged 40 to 59 years with 16.9%, individuals aged 75 years and above with 6.8%, and those aged 60 to 74 years with 6.3%.

**Figure 4.**
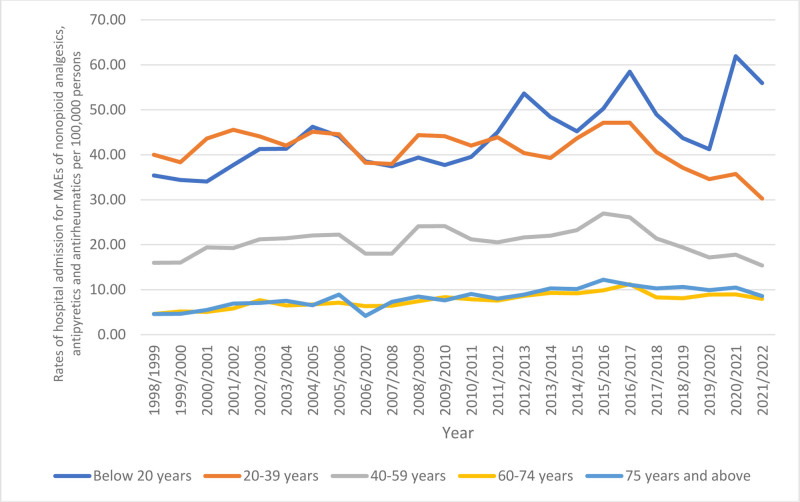
Admission rate stratified by age group.

Rates of admission for MAEs among patients aged below 20 years increased by 58.2% from 35.38 (95% CI 33.77–37.00) in 1998 to 55.97 (95% CI 54.12–57.82) in 2022 per 1,00,000 persons. Conversely, rates of admission among patients aged 20 to 39 years decreased by 24.3% from 40.01 (95% CI 38.36–41.66) in 1998 to 30.27 (95% CI 29.00–31.53) in 2022 per 1,00,000 persons. Furthermore, rates of admission for MAEs among patients aged 40 to 59 years decreased by 3.8% from 15.99 (95% CI 14.86–17.11) in 1998 to 15.38 (95% CI 14.43–16.33) in 2022 per 1,00,000 persons. However, rates of admission for MAEs among patients aged 60 to 74 years increased by 72.6% from 4.60 (95% CI 3.68–5.53) in 1998 to 7.94 (95% CI 7.06–8.82) in 2022 per 1,00,000 persons. Similarly, rates of admission for MAEs among patients aged 75 years and above increased by 86.1% from 4.60 (95% CI 3.29–5.92) in 1998 to 8.56 (95% CI 7.28–9.85) in 2022 per 1,00,000 persons (Fig. 4).

### 3.5. Trends in admission stratified by gender

**Figure 5.**
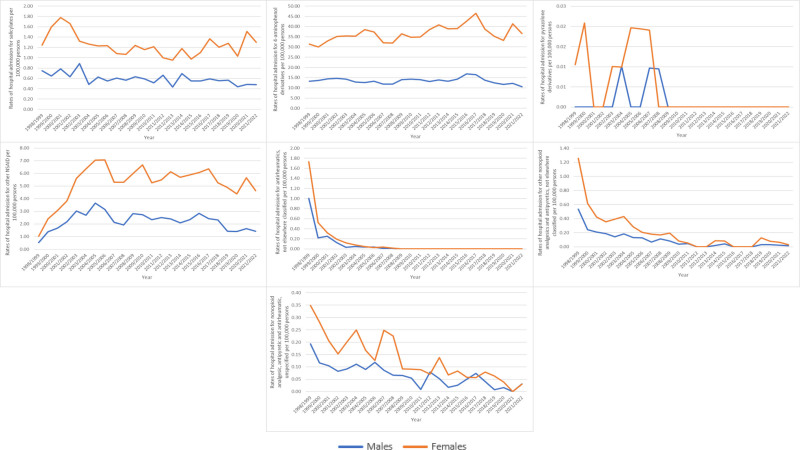
Admission rates stratified by gender and indication.

Throughout the study period, hospital admission rates for MAEs of nonopioid analgesics, antipyretics and antirheumatics were higher among females compared to males (*P* < .001; Fig. 5).

### 3.6. Trends in admission stratified by age

**Figure 6.**
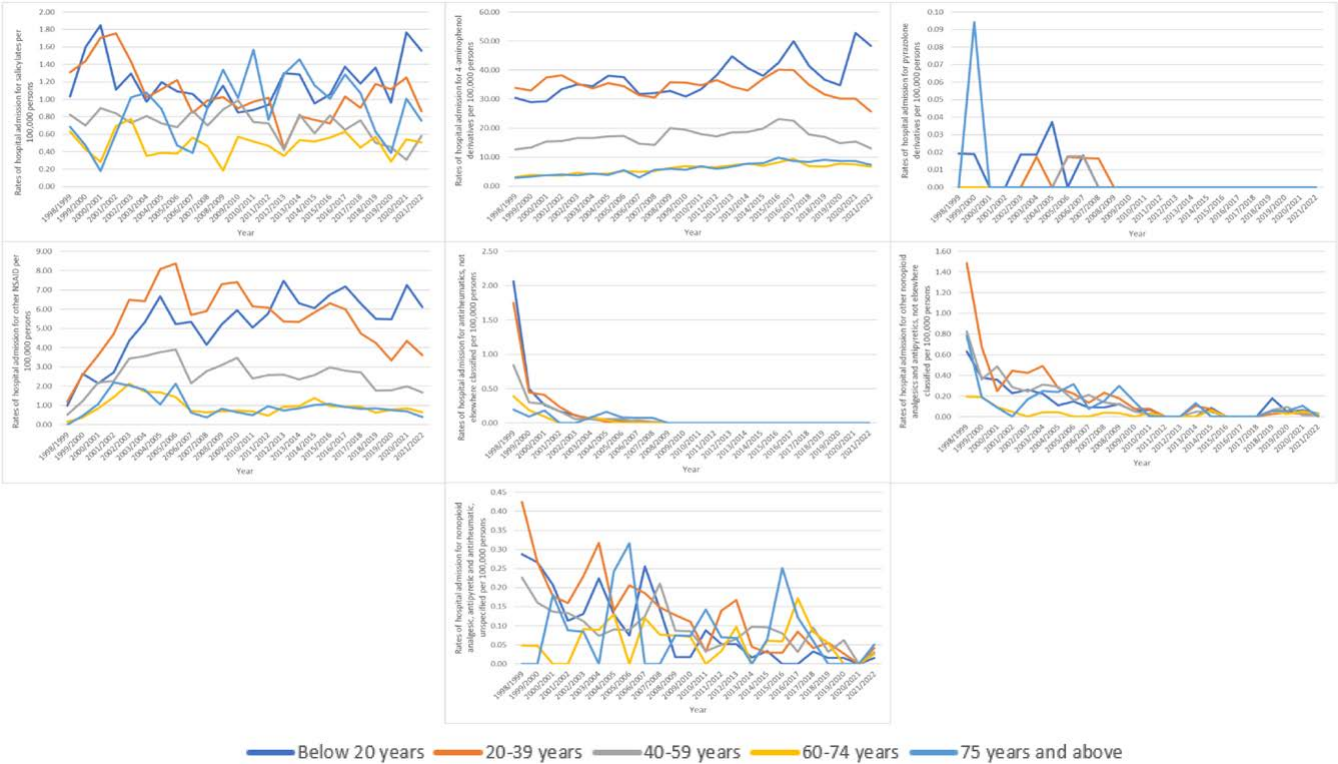
Admission rates stratified by age group and indication.

The majority of admission rates for MAEs were observed in the age group below 20 years. That includes the following: salicylates, 4-aminophenol derivatives, pyrazolone derivatives, and “antirheumatics, not elsewhere classified.” Still, hospital admission rates for MAEs of other NSAIDs, “other nonopioid analgesics and antipyretics, not elsewhere classified,” and “nonopioid analgesic, antipyretic and antirheumatic, unspecified” were more prevalent among individuals aged 20 to 39 years (Fig. 6).

## 4. Discussion

Nonopioid analgesics, such as acetaminophen (paracetamol) and NSAIDs, are commonly utilized as OTC antipyretics and analgesics.^[21,22]^ The utilization of nonopioids increases concerns due to their potential for serious side effects, including raised risk of intoxication, fetotoxicity, and severe infectious or renal problems.^[23–26]^ For instance, although paracetamol is commonly considered safe within therapeutic limits, it can lead to considerable morbidities, such as liver injury,^[27–29]^ and may lead to death in cases of overdose.^[28–30]^ In Denmark, the risk of suicide later in life was found to be considerably increased by previous hospital admissions for poisoning caused by nonopioid analgesics.^[31]^

Our study revealed a 43.6% increase in the overall annual number of poisonings related to nonopioid analgesics, antipyretics, and antirheumatics, resulting in a rise of 3.8% in hospital admission rates for various causes. One possible explanation for this could be the extensive utilization of OTC medications in Australia.^[15,32–34]^ The wide availability of OTC can result in dependence, misuse,^[35]^ unintentional ingestion, intentional self-harm, and fatal consequences in particular cases.^[36–40]^ Our finding is in line with the rise of hospital admission rates for poisonings related to nonopioid analgesics, antipyretics, and antirheumatics in the Lower Silesia region of Poland from 2006 to 2012,^[41]^ among pediatrics in England and Wales from 1999 to 2020,^[42–44]^ among the total population in England and Wales from 1999 to 2020.^[8]^ These findings highlight the need for targeted interventions to raise awareness of the risks associated with nonopioid analgesics, antipyretics, and antirheumatic drugs, and thus reduce toxicity associated with these medications.

Our study revealed that overnight-stay of hospitalizations due to MAEs of nonopioid analgesics, antipyretics, and antirheumatics comprised 71.0% of the total cases, whereas 29.0% were same-day admissions. Still, same-day admissions for these drugs decreased by 12.3%, while overnight admissions increased by 11.7%. The majority of overnight admissions compared to same-day admissions indicates the seriousness of MAEs of nonopioid analgesics, antipyretics, and antirheumatics, which may require prolonged hospitalization for monitoring, treatment, and observation of potential complications. Other factors that could contribute to lengthy hospital admissions include age 5 to 14 years and female sex.^[45,46]^

According to our study, MAEs of 4-aminophenol derivatives appeared as the most common cause of hospital admissions related to nonopioid analgesics, antipyretics, and antirheumatics, comprising 83.5% of the total cases, followed by MAEs of other NSAIDs at 12.4%. Moreover, a significant 2.91-fold increase in hospital admission rates due to poisoning with other NSAIDs was observed. Besides, hospital admission rates for MAEs of 4-aminophenol derivatives increased by 6.0%. These findings are consistent with several previous investigations. A prior study in Australia declared that there was a 44% increase in the yearly number of hospital admissions related to paracetamol poisoning from 2007/2008 to 2016/2017.^[47]^ A study conducted among pediatric patients in England and Wales identified 4-aminophenol derivatives (like paracetamol) and other NSAIDs as the primary causes of hospital admissions related to nonopioid analgesics, antipyretics, and antirheumatics.^[42]^ Moreover, an earlier report from Australia demonstrated that the bulk of accidental poisonings and exposures to nonopioid analgesics, antipyretics, and antirheumatics were attributed to 4-aminophenol derivatives.^[48]^ Similarly, in Spain, Australia, Denmark, France, and the UK, paracetamol stands out as the most commonly used nonopioid analgesic.^[23,49]^ In contrast, in Germany, ibuprofen is the predominant nonopioid analgesic.^[23,50]^ These underscore the variability in nonopioid analgesic utilization practices across different European countries. Again, these findings emphasize the necessity of raising awareness of the risks of paracetamol and other NSAIDs by applying targeted interventions.

One of the primary reasons for poisoning in young children is medication administration errors related to nonopioid analgesics, antipyretics, and antirheumatics.^[51]^ The result of our study indicates that hospital admission rates for MAEs of nonopioid analgesics, antipyretics, and antirheumatics were higher among individuals below the age of 20 years. Notably, rates of hospital admissions for MAEs of these medications in patients aged below 20 years increased by 58.2%. These results align with numerous prior studies. Every day, about ten children in Australia are hospitalized due to poisoning.^[3]^ Pharmaceutical substances were responsible for more than 70% of these cases; non-opioid analgesics, antipyretics, and antirheumatics accounted for the most cases (37.1%).^[3]^ In British Columbia hospitals, the most prevalent substances used for self-poisoning among those aged 10 to 19 years were nonopioid analgesics, antipyretics, and antirheumatics.^[52]^ In the United States, 37.3% of poisoning-related attempted suicides were due to OTC analgesics that resulted in serious consequences among the age group 10 to 25 years.^[53]^ This raises concerns about the elevated self-poisoning rates utilizing nonopioid analgesics, antipyretics, and antirheumatics.^[53]^ Furthermore, between 2006 and 2016, there were 8.4% yearly increases in intentional poisonings in patients aged from 5 to 19 years in the Australian states of New South Wales and Victoria.^[54]^ These imply a worrying trend in intentional poisonings in this age range and underscore the global scope of nonopioid analgesics, antipyretics, and antirheumatics’ role in pediatric poisoning incidents, as well as the importance of addressing it through comprehensive strategies.

Consistent with a previous report in Australia,^[48]^ our study revealed higher hospital admission rates among females than males for MAEs of nonopioid analgesics, antipyretics, and antirheumatics. Previous research indicates that females exhibit a higher poisoning susceptibility than males, which could interpret our findings.^[55–57]^ Furthermore, findings from Brazil indicate that adults and women had more elevated rates of hospital admissions and poisoning after paracetamol exposures.^[58]^ This observation may suggest a gender disparity in coping strategies, with women potentially leaning toward utilizing drug overdose as a method of self-harm, in contrast to men who could lean towards physical self-harm practices.^[59]^ Supporting by evidence from various nations indicates that women are more prone to practice self-harming behaviors.^[60]^ However, the suicide mortality rate is lower for men than women.^[60]^ Furthermore, exposure to medication non-adherence, dosing errors, self-harm attempts, and polypharmacy are among the additional aspects that play a significant role in broadening the disparity between males and females and raising toxicity levels.^[61]^ These emphasize the critical need for targeted interventions to address the elevated vulnerability of females to poisoning and improve healthcare outcomes for this demographic.

There are limitations on this study. The present study was limited in its ability to examine patient-level data and investigate the underlying causes of the observed patterns due to the constraints associated with publicly accessible web-based information. Due to the nature of publicly available data, this analysis could not account for factors including lifestyles, comorbidities, polypharmacy, or demography, making it difficult to control confounding variables. The overestimation of the hospital admissions rate may have resulted from the inclusion of repeated hospitalization episodes for the same patients in the hospitalization data.

## 5. Conclusion

Medication administration errors of nonopioid analgesics, antipyretics, and antirheumatics-related hospitalizations rose significantly in Australia between 1998 and 2022. Overnight stays accounted for most hospital admissions episodes, indicating high-risk situations requiring long-term follow-up. Medication administration errors of 4-aminophenol and other NSAIDs were the most common reasons for admission. Hospital admissions for other NSAID MAEs increased significantly. Hospital admission trends vary across age groups and genders (age <20 years and female gender showed higher hospital admissions). Increasing public awareness about medication risks, especially other NSAIDs is critical. Moreover, targeted interventions are necessary to reduce the risks of use and hospitalizations associated with these medications, particularly among females and young populations.

## Acknowledgments

We would like to acknowledge Princess Nourah bint Abdulrahman University Researchers Supporting Project number (PNURSP2025R483), Princess Nourah bint Abdulrahman University, Riyadh, Saudi Arabia.

## Author contributions

**Conceptualization:** Asaleh El-Qasem, Abdallah Y. Naser.

**Data curation:** Asaleh El-Qasem, Abdallah Y. Naser, Alaa A. Alsharif.

**Formal analysis:** Abdallah Y. Naser.

**Investigation:** Asaleh El-Qasem, Abdallah Y. Naser, Alaa A. Alsharif.

**Methodology:** Asaleh El-Qasem, Abdallah Y. Naser.

**Project administration:** Abdallah Y. Naser.

**Resources:** Asaleh El-Qasem, Abdallah Y. Naser, Alaa A. Alsharif.

**Software:** Abdallah Y. Naser.

**Supervision:** Abdallah Y. Naser.

**Validation:** Asaleh El-Qasem, Abdallah Y. Naser, Alaa A. Alsharif.

**Visualization:** Asaleh El-Qasem, Abdallah Y. Naser.

**Writing – original draft:** Asaleh El-Qasem, Abdallah Y. Naser, Alaa A. Alsharif.

**Writing – review & editing:** Asaleh El-Qasem, Abdallah Y. Naser, Alaa A. Alsharif.
